# Physical activity behaviour change in people living with and beyond cancer following an exercise intervention: a systematic review

**DOI:** 10.1007/s11764-023-01377-2

**Published:** 2023-04-19

**Authors:** Chloe E. Salisbury, Melissa K. Hyde, Ella T. Cooper, Rebecca C. Stennett, Sjaan R. Gomersall, Tina L. Skinner

**Affiliations:** 1 School of Human Movement and Nutrition Sciences, The University of Queensland, Brisbane, QLD Australia; 2 School of Psychology, The University of Queensland, Brisbane, QLD Australia; 3 School of Health and Rehabilitation Sciences, The University of Queensland, Brisbane, QLD Australia

**Keywords:** Systematic review, Physical activity, Exercise, Cancer, Behaviour change, Behaviour change techniques, Maintenance

## Abstract

**Purpose:**

Exercise interventions can increase physical activity and wellbeing of people living with/beyond cancer. However, little is known about maintenance of physical activity in this population ≥ 6 months post-exercise intervention, when theoretical evidence suggests behaviour maintenance occurs. Study aims are to (i) systematically review maintenance of physical activity ≥ 6-month post-exercise intervention, and (ii) investigate the influence of behaviour change techniques (BCTs) on physical activity maintenance in people living with/beyond cancer.

**Methods:**

CINAHL, CENTRAL, EMBASE and PubMed databases were searched for randomised controlled trials up to August 2021. Trials including adults diagnosed with cancer that assessed physical activity ≥ 6 months post-exercise intervention were included.

**Results:**

Of 142 articles assessed, 21 reporting on 18 trials involving 3538 participants were eligible. Five (21%) reported significantly higher physical activity ≥ 6 months post-exercise intervention versus a control/comparison group. Total number of BCTs (M = 8, range 2–13) did not influence intervention effectiveness. The BCTs Social support, Goal setting (behaviour), and Action planning, alongside supervised exercise, were important, but not sufficient, components for long-term physical activity maintenance.

**Conclusions:**

Evidence for long-term physical activity maintenance post-exercise intervention for people living with/beyond cancer is limited and inconclusive. Further research is required to ensure the physical activity and health benefits of exercise interventions do not quickly become obsolete.

**Implications for Cancer Survivors:**

Implementation of the BCTs Social support, Goal setting (behaviour), and Action planning, alongside supervised exercise, may enhance physical activity maintenance and subsequent health outcomes in people living with/beyond cancer.

**Supplementary Information:**

The online version contains supplementary material available at 10.1007/s11764-023-01377-2.

## Introduction

Approximately 19.3 million new cases of cancer were diagnosed worldwide in 2020 [1], with incidence expected to rise to 21.7 billion by 2030 [2]. Modern cancer treatments exhibit high rates of success [3]; however, their physical and psychological side effects often result in long-term health concerns following treatment completion [4, 5]. A wealth of evidence supports the efficacy of physical activity as a non-pharmacological adjuvant therapy for preventing and/or alleviating disease- and treatment-related side effects, including cancer-related fatigue, physical functioning, and psychological distress [4, 6]. However, physical activity levels decline following a cancer diagnosis [7], with the vast majority of people with cancer insufficiently active to achieve health benefits [8].

Structured exercise interventions are an effective means of increasing physical activity in people with cancer [4, 9, 10]. Systematic reviews demonstrate high physical activity adherence rates (70–86%) in people with cancer during these interventions [9, 11, 12]. Further, Bluethmann et al. [13] found in a systematic reviews and meta-analyses of 14 interventions in people living beyond breast cancer that exercise interventions were successful at producing short-term physical activity levels. However, these levels are often not maintained beyond the duration of the intervention [11, 13] and the long-term maintenance of physical activity levels following completion of an exercise intervention in people with cancer is less clear.

Grimmett et al. [14] noted in a systematic review and meta-analysis that people with cancer maintained their physical activity levels for at least three months following exercise or multimodal health interventions targeting aerobic physical activity (SMD = 0.25; *p* < 0.01); a small positive effect was maintained when isolating studies ≥ 6 months follow-up post-intervention (SMD = 0.21; *p* < 0.001). However, Grimmett et al. [14] included multimodal interventions (e.g. exercise and nutrition) and did not isolate the findings from exercise interventions alone, and only articles that reported physical activity as moderate-to-vigorous minutes per week were included in the meta-analysis, excluding 30% of eligible articles from the analysis that reported physical activity by other means (e.g. MET/week, walking time). Contrary to Grimmett et al.’s [14] results, Spark et al. [15] noted only three trials (30%) achieved successful maintenance of physical activity ≥ 3 months post-intervention in a systematic review of physical activity and/or dietary interventions in people living with and beyond breast cancer. Further, Finlay et al. [16] conducted a systematic review on physical activity maintenance in people living with and beyond prostate cancer, and reported physical activity maintenance at 3–6 months follow-up in only two (17%) of the included articles, with only one of those trials also demonstrating maintenance > 6 months. Research to date has focused on physical activity levels between 3 and 6 months post-intervention [14–16]; however, according to the transtheoretical model, ≥ 6 months of follow-up is required to confirm maintenance of behaviour change [17], as people in this stage are less tempted to relapse, with increased confidence they can sustain their new behaviour. Thus, long-term maintenance of physical activity following an exercise intervention in people with cancer remains a novel and salient area of investigation as without long-term maintenance of these behaviours, the short-term health benefits quickly become diminished.

Maintenance of physical activity is a multifaceted process, with individuals facing varying needs and challenges in sustaining their activity levels over time [18]. Behavioural science can provide important insight into physical activity maintenance through understanding the psychological, environmental, and social factors that influence human behaviour, including physical activity behaviour [19, 20]. Designing interventions with consideration of these behavioural factors may influence the effectiveness in promoting long-term physical activity maintenance in people with cancer. Behaviour change techniques (BCTs) are distinct components of an intervention that help change or adjust the processes that regulate behaviour [21], such as participation in physical activity. The BCT taxonomy is a framework used to help standardise the reporting of behaviour change interventions and consists of 93 BCTs that represent observable, replicable, and irreducible components of an intervention aimed at altering behaviour [21]. Grimmett et al.’s [14] systematic review was the first to use the BCT Taxonomy v1 [21] to identify and classify BCTs present in interventions for all oncological populations with a post-intervention follow-up, and noted that unsuccessful interventions were less likely to include Social Support (Unspecified), Action Planning, and Graded tasks. Similarities were noted between BCTs within included articles with statistically significant between-group differences, within-group differences, and those with neither between- nor within-group differences at post-intervention follow-up, making it difficult to identify BCTs that were most effective [14]. However, the authors did not distinguish the difference in BCTs used in interventions with maintenance of physical activity at a follow-up of 3-months compared with 6 months. Evidence in healthy adults indicates that BCTs effective in the short-term (< 6 months) versus long-term (≥ 6 month) maintenance of physical activity can differ [22], and collating interventions with ≥ 3 months follow-up means that this overlap may make it difficult to identify the BCTs that are effective for long-term behaviour change.

The primary aim of this paper is to extend the work of Grimmett et al. [14] by systematically reviewing the available literature exploring the long-term (≥ 6 months) maintenance of physical activity following the completion of an exercise intervention compared to a control/ comparison group in individuals with a histologically confirmed diagnosis of cancer. A secondary aim was to use the BCT taxonomy (version 1) [21] to identify intervention components that may influence long-term physical activity maintenance following an exercise intervention in people living with and beyond cancer.

## Materials and methods

### Search strategy

This systematic review was conducted according to the Preferred Reporting Items for Systematic review and Meta-Analysis (PRISMA) statement [23]. From the earliest time point available to August 2021, four key databases were systematically searched: CINAHL, CENTRAL (Cochrane Central Register of Controlled Trials), EMBASE and PubMed. Where possible, search terms were developed using index (PubMed [MeSH]) and thesaurus terms (PubMed, CENTRAL [tiab], and EMBASE [EMtree]). Notwithstanding, free-text population terms were used to generate search functions according to the inclusion criteria. These were amalgamated with Boolean operators and truncation functions. Free terms for exercise ("exercise", "resistance training", exercis*, "physical activity", "weight training", resistance, strength, endurance, aerobic) were used in AND-combination with search terms identifying the target population (“neoplasms”, neoplasm*, cancer, carcinoma) and specific trial design (program*, intervention*). Where possible, filters were used to refine the search to include only human clinical trials published in the English language and in peer-reviewed journals, without the use of external limiters. A complete list of search terms is available upon request (an example search can be found in supplementary materials).

### Article selection

The inclusion criteria were specified by the Population, Intervention, Control, Outcomes, Study design (PICOS) framework. This included the following:

(i) Population: adults aged 18 years and older with a histologically confirmed diagnosis of cancer (including all stages of, and treatments for, cancer);
(ii) Intervention: any structured aerobic- and/or resistance-exercise based intervention where specific exercise and/or physical activity advice was provided to participants (interventions limited to specific areas of the body, such as pelvic floor exercises, and multimodal interventions were excluded);
(iii) Control: groups receiving usual or standard care; groups not receiving exercise and/or physical activity advice; groups receiving different exercise and/or physical activity advice; and, groups receiving the same initial exercise and/or physical activity advice with a different type, frequency or intensity of support in the follow-up period;
(iv) Outcome: any measure of physical activity ≥ 6 months following the completion of the primary exercise intervention;
(v) Study design: randomised controlled trials (RCT). Only English, full-text articles of human trials published in peer-reviewed journals were included.

Title and abstract screening was performed independently by C.S. and E.C. to exclude articles outside the scope of this review. Two authors (C.S. and E.C.) completed independent assessments of the remaining full-text articles for eligibility according to the inclusion criteria. Disagreements were resolved by discussion until consensus was reached. If consensus could not be reached, a third author (T.S.) acted as the arbiter. Reference lists were searched manually to identify additional eligible articles.

### Data extraction and analysis

Article details were extracted and collated for analysis by C.S., R.S., and E.C. Extracted information included author/s, year, inclusion and exclusion criteria, trial design, sample size, and description of the intervention and/or control groups. Participant characteristics included cancer type, age, gender, and if participants were undergoing cancer treatment/s during the trial. Intervention details recorded were frequency, intensity, duration and mode of intervention, supervision, type of delivery, theoretical basis of behaviour change, BCTs reported, and length of follow-up. The follow-up period was defined as the time immediately succeeding the exercise intervention to the final follow-up testing. Data extraction also included adherence and attendance to the intervention and trial drop-out rate at follow-up. Physical activity outcome data including the method used to measure physical activity, all absolute or relative change and change scores, and significance testing were extracted. In instances where data were presented graphically [24, 25], data were extracted via *graphreader* software (http://www.graphreader.com). In cases where results were not clear, C.S., E.C., and R.S. discussed the item to reach consensus.

### Quality assessment

Quality assessment was conducted using Cochrane Collaboration’s tool for assessing risk of bias [26], developed specifically for randomised controlled trials. The tool features seven criteria for assessing the risk of bias in various methodological aspects. Each criterion was rated using a ‘low risk of bias’ (L; 1 point), ‘some concerns of bias’ (U; 0 points), or ‘high risk of bias’ (H; 0 points). A final quality score was determined as the total number of articles scoring a point in each category divided by the total number of articles. Quality assessment was performed independently by two authors (C.S., and E.C.) and final decisions were reached through discussion and consensus. Meta-analysis was not performed in this review due to the heterogeneity of the population, intervention, and physical activity measures.

### Coding of behaviour change techniques

The BCT taxonomy version 1 [21] was used to identify and code the BCTs reported in each intervention group. The target behaviour of the BCTs was physical activity (e.g. daily walking), and the target population was people living with or beyond cancer. The target outcome of this review was overall physical activity levels (e.g. MET-h/week or moderate-to-vigorous minutes per week). Coding was carried out by C.S. and E.C. independently after completing the BCT taxonomy version 1 Online Training [27] by using the provided BCT definitions and coding rules. BCTs were coded as present or absent, and only BCTs exclusively applied in the intervention group/s were extracted. BCTs were coded based on the information presented in the included papers, in addition to any published protocol papers or published papers of the same trial. The first five articles were coded independently, and the authors compared, discussed, and clarified additional coding rules to interpret ambiguities. Discrepancies in coding were resolved through discussion, by referring to the taxonomy and consulting with a third author (M.H.).

To assess intercoder agreement, prevalent-adjusted bias-adjusted kappa (PABAK) [28] was used based on the semi-final coding. PABAK was chosen as it adjusts for potential chance agreement between coders and high prevalence of negative agreement (i.e. when both coders agree the BCT is absent). Where both coders identified the BCT as present or absent, agreement was recorded and where one coder identified the BCT, but the other coder did not identify the BCT, disagreement was recorded. PABAK was calculated for each of the BCTs, with a good reliability considered as a score of 0.60 or above.

## Results

### Identification and selection of articles

**Fig. 1:**
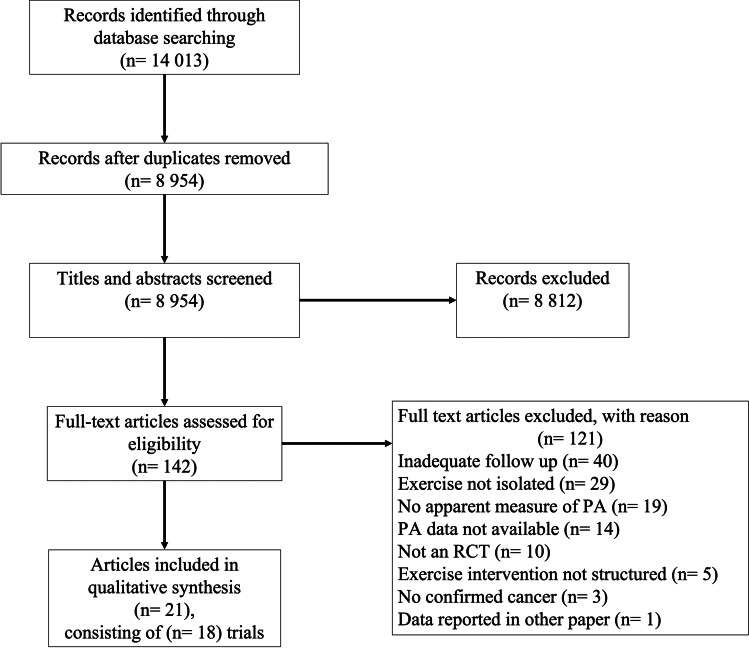
CONSORT diagram of literature search

Details of the systematic search process are outlined in Fig. 1. A total of 14,013 articles were retrieved from a combination of database search results. Following both automatic (Covidence, www.covidence.org) and manual (C.S.) removal of duplicates, 8954 were screened for title and abstract. Full texts of 142 articles were retrieved and assessed. Following agreement among all authors, 21 [24, 25, 29–47] met the inclusion criteria and were included in the qualitative synthesis. The 21 articles reviewed reported on 18 individual trials. Two ([38, 39] and [24, 31]) of the individual trials published in more than one article reported results at different follow-up timeframes, whilst the other [40, 46] reported results on different subsamples of participants.

### Quality assessment

**Fig. 2:**
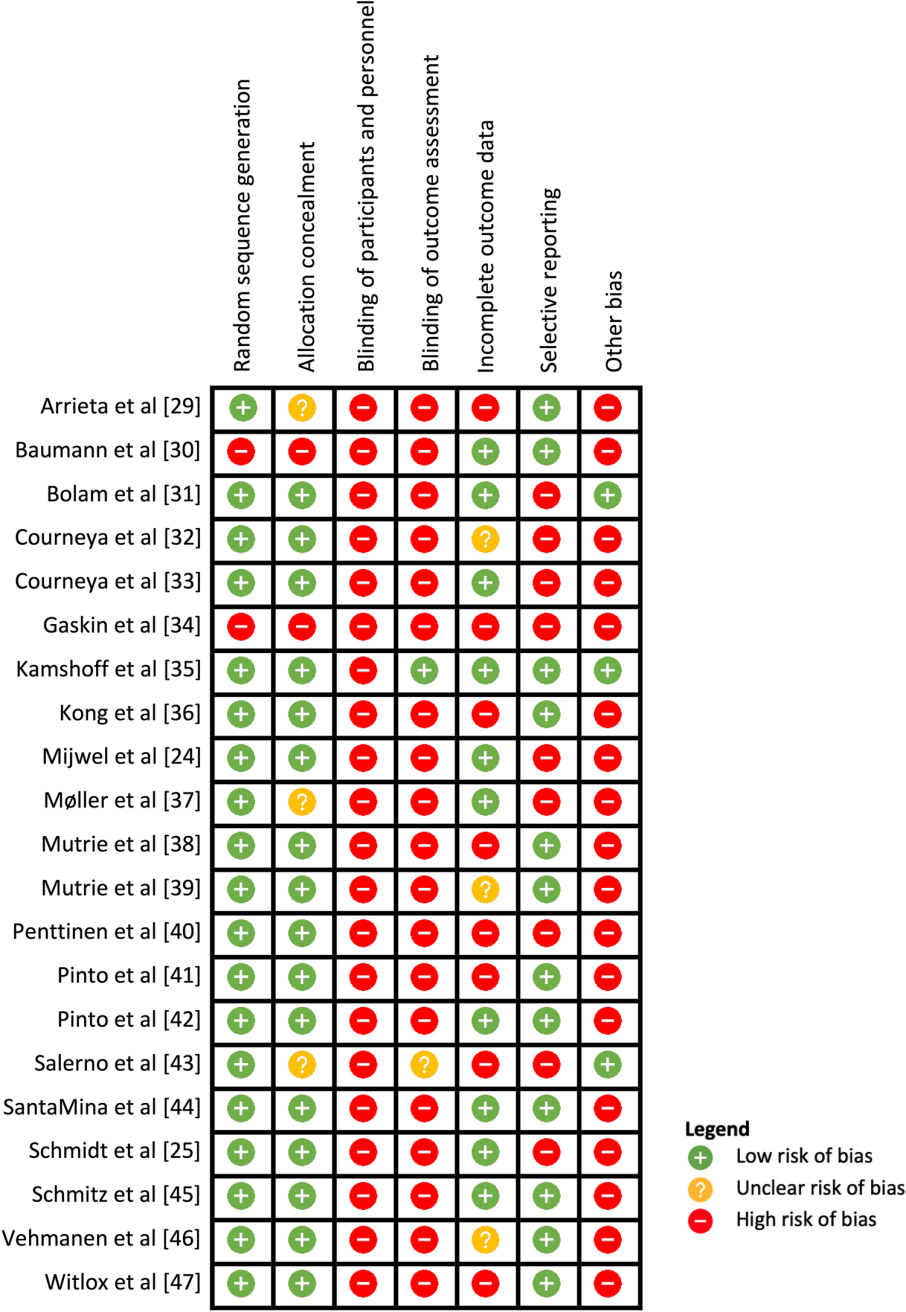
Risk of bias analysis using the Cochrane Collaboration’s risk of bias tool

Risk of bias ratings for the 21 included articles are presented in Fig. 2. The mean quality score for the included articles was 40%, with scores ranging from 0% [34] to 86% [35]. Of note, assessor blinding for the main outcome (physical activity) was completed by participant self-report in 17 articles, so blinding was not possible [24, 25, 29, 30, 32, 34, 36–42, 44–47]. In three articles where blinding was possible due to a device-based assessment method, assessors were blinded in one study [35], not blinded in another [31], and the third article did not state if the outcome assessor was blinded [43]. There was a high risk of bias from other sources for 17 articles [24, 25, 29, 30, 32, 34, 36–42, 44–47] that used a self-reported physical activity measure, which is prone to bias.

### Participant characteristics

Characteristics of the participants in the trials included in this review are described in Table 1. A total of 3538 participants (78% female) were included in the 18 trials; sample sizes ranged from *n* = 46 [42, 43] to *n* = 573 [40, 46] (median *n* = 193). Participants were an average of 58 years of age across all trials (range 18–82 years). Ten [24, 25, 30–32, 36–41, 45, 46] of the 18 trials exclusively investigated women with breast cancer; the remaining trials involved participants with prostate cancer (*n* = 2) [34, 44], colorectal cancer (*n* = 1) [42], lymphoma (*n* = 1) [33], breast or colorectal cancer (*n* = 1) [47], and breast, colorectal or other cancer (*n* = 3) [29, 35, 43]. Participants were undergoing anti-cancer treatment in nine trials [24, 25, 29, 31, 32, 36–39, 44, 47], had completed treatment in eight trials [30, 34, 35, 40–43, 45, 46], with one trial including participants both undergoing treatment and post-treatment [33].

**Table 1:** Overview of participants and interventions

Reference	n	Cancer type	On treatment	Mean age	% Female	Intervention provider	Setting/ mode of delivery	Intervention length	Control group	Outcome measure	Follow-up^a^	Within-group difference	Between-group difference
Arrieta et al. [29]	301	Breast, Colon, Other	Yes	76.7	60	Professional instructor with degree in PA and sport sciences	Unsupervised phone-based multimodal program	12 months	Standard PA recommendations	Self-report (IPAQ)	18 and 24 months	NS	NS
Baumann et al. [30]	194	Breast	No	55.85^b^	100	Unclear	Program of participant preference	3 weeks	Usual care (3-week program of German rehabilitation guidelines)	Self-report (FFkaA)	8, 12^c^, 18, and 24^c,d^ months	Sig.^c^	Sig.^d^
Bolam et al. [31] & Mijwel et al. [24]	206	Breast	Yes	53.2^b^	100	Exercise physiologist or oncology nurse	Supervised AET + HIIT or RET + HIIT program	16 weeks	Standard PA recommendations	Device-based (accelerometry)	2 years	NS	NS
										Self-report (single item question)	12 months	NS	NS
Courneya et al. [33]	122	HL or NHL	Mixed	53.2	41	Exercise physiologist	Supervised AET program	12 weeks	Maintain previous PA levels. Offered 4-week supervised exercise post-IV	NR	9 months	NR	Sig
Courneya et al. [32]	242	Breast	Yes	49.2	100	Unclear	Supervised AET or RET program	Median = 17 weeks (95% CI, 9 to 24 weeks)	Asked not to initiate any PA. Offered a 1-month exercise program post-IV	Self-report (GLTEQ)	6 months post-IV	NS	NS
Gaskin et al. [34]	147	Prostate	No	66.0	0	Exercise physiologist	Supervised and unsupervised multimodal intervention	12 weeks	Usual care	Self-report (GLTEQ)^e^	12 months	NR	NS
Kampshoffet al [35]	277	Various (Breast 65.5%^b^)	No	53.5^b^	80^b^	Physiotherapist	Supervised LMI AET and RET or HI AET and RET program	12 weeks	Waitlist control	Device-based (accelerometry)	15 months	NS	NS
Kong et al. [36]	152	Breast	Yes	47.0	100	Exercise physiologist	Unsupervised, PA counselling + WAT or PA counselling only	5 weeks	NA	Self-report (GPAQ)^e^	29 weeks	NS	NS
Møller et al. [37]	153	Breast	Yes	51.7	100	Exercise physiologist/physical therapist + clinical nurse specialist or clinical nurse specialist	Supervised multimodal program or unsupervised walking program	12 weeks	NA	Self-report (self-developed)	9 months	Sig	Sig
Mutrie et al. [38] & Mutrie et al. [39]	203	Breast	Yes	51.6	100	Exercise specialist	Supervised AET and RET program	12 weeks	Standard PA recommendations	Self-report (SPAQ)	21 and 63^d^ months	NS	Sig.^d^
											9 months	NS	NS
Penttinen et al. [40]	573	Breast	No	52.35^b^	100	Physical therapist	Supervised group-based step AET and circuit training + unsupervised home AET	12 months	Maintain previous PA levels	Self-report (prospective 2-week PA diary)	5 years	NR	NS
Vehmanenet al [46]		Breast		51.9^b^								NS	NS^f^
Pinto et al. [41]	192	Breast	No	60.0	100	NR	Health advice + unsupervised AET program (telephone counselling)	12 weeks	Health advice + contact control	Self-report (7-day PAR)^e^	12 months	Sig	NS
Pinto et al. [42]	46	Colorectal	No	57.3	56	NR	Unsupervised PA program (telephone counselling)	12 weeks	Contact control	Self-report (7-day PAR)^e^	12 months	Sig	NS
Salerno et al. [43]	46	Various (Breast 37%)	No	72.9	80.4	NA	Home DVD-based multimodal program + telephone calls	6 months	Standard PA recommendations + contact control	Device-based (accelerometry)	12 months	NS	NS
SantaMina et al. [44]	66	Prostate	Yes	71.35^b^	0	Exercise physiologist	Home-based, AET^g^ or RET program	6 months	NA	Self-report (GLTEQ)	12 months	Sig.^g^	NS
Schmidt et al. [25]	227	Breast	Yes	54.6	100	Unclear	Supervised RET program	12 weeks	Progressive muscle relaxation. Offered 12wk RET post-IV	Self-report (adapted SQUASH)^e^	15 months	NS	NS
Schmitz et al. [45]	154	Breast	No	55^b^	100	Fitness professional	Supervised group based RET program	13 weeks	Maintain previous PA levels	Self-report (IPAQ)	12 months	NS	NS
Witlox et al. [47]	237	Breast, Colon	Yes	50.75^b^	91.2^b^	Physiotherapist	Supervised AET and RET program	18 weeks	Usual care	Self-report (SQUASH)	4 years	Sig	Sig

*PA* physical activity, *AET* aerobic exercise training, *RET* resistance exercise training, *HIIT* high intensity interval training, *LMI* low-to-moderate intensity, *HI* high intensity, *HL* Hodgkin lymphoma, *NHL* non-Hodgkin lymphoma, *IPAQ* International Physical Activity Questionnaire, *FFkaA* Freiburg questionnaire on physical activity, *GLTEQ* Godin Leisure-Time Physical Activity Questionnaire, *GPAQ* Global Physical Activity Questionnaire; *SPAQ* Scottish Physical Activity Questionnaire, *PAR* Physical Activity Recall Scale, *SQUASH* Short Questionnaire to Assess Health-Enhancing Physical Activity, *Sig*. significant, *NS* not-significant, *NR* not reported, *NA* not applicable. ^a^from baseline; ^b^summed means across groups; ^c^within-group changes at the respective timepoint only; ^d^between-group changes at the respective timepoint only; ^e^physical activity was the primary outcome for the trial; ^f^comparison of pre-menopausal and post-menopausal participants; ^g^within-group changes in the respective group only

### Intervention characteristics

A brief overview of the intervention characteristics of the trials is described in Table 1, with further details described in Table 2. Further details on the intervention details can be found in supplementary materials. The length of exercise interventions ranged from 3 weeks [30] to 52 weeks [29, 40, 46], with a mean of 17.2 weeks.

**Table 2:** Study intervention details

Reference	G	Sup	Frequency	Intensity	Time	Mode	Other Rec	Adh	Att	Intervention length	Dropout^a^
Arrieta et al. [29]	IG	No	2/wk	Low–high	NR RET: 10 reps progressing	AET, RET (UL, LL), balance, proprioception, flexibility	-	NR	70.1%	12 months	18 months 19% 24 months 43%
	CG	-	Standard PA recommendations				-	-	-		18 months 15% 24 months 34%
Baumann et al. [30]	IG	NR	15METs/wk	Preferred sports activity (or treadmill, walking, machine weight training)	-		-	NR	NR	3 weeks	NR
	CG	-	Usual care (3- week program of German rehabilitation guidelines)				-	-	-		NR
Bolam et al. [31] & Mijwel et al. [24]	IG1	Yes	2/wk	HIIT: RPE 16–18 AET: RPE 13–15	HIIT: 3 × 3 min (1 min rest) AET: 20 min (60 min total)	HIIT: cycle ergometer AET: cycle ergometer, elliptical ergometer, or treadmill	-	75%	63%	16 weeks	2 years 33% 12 months 18%
	IG2	Yes	2/wk	HIIT: RPE 16–18 RET: Initially 70% estimated 1RM, increasing to 80% estimated 1RM when > 12repetitions achieved	HIIT: 3 × 3 min (1 min rest) RET: 2 sets 8–12 reps, (60 min total)	HIIT: Cycle ergometer RET: Machine and free weights (8 exercises targeting UL, LL and core)	-	83%	68%		2 years 27% 12 months 16%
	CG	-	Usual care (general information on physical activity)				-	-	-		2 years 54% 12 months 13%
Courneya et al. [33]	IG	Yes	3/wk	wk1 @ 60% PPO; wk2 @ 65% PPO; wk3 @ 70%; wk4 + @ 75% PPO (wk7 + 1/wk @ above VT & wk9 @ 1 × V02peak interval)	15-20 min (wk 1–4), increasing by 5 min to 40-45 min (from wk 9)	AET: upright or recumbent cycle ergometer	-	Duration: 99% Intensity: 90.7%	77.8%	12 weeks	8%
	CG	-	Asked not to increase exercise above baseline. Offered 4-week supervised exercise post-intervention assessments (42% attendance)				-	-	-		11%
Courneya et al. [32]	IG1	Yes	3/wk	60% VO_2peak_ progressing to 80% VO_2peak_	15 min progressing to 45 min	AET: cycle ergometer, treadmill, or elliptical trainer	-	Duration 95.6% Intensity 87.2%	72%	Median: 17 weeks (9–24 weeks)	13%
	IG2	Yes	3/wk	60–70% estimated 1RM, progressing weight by 10% when > 12reps could be completed	2 sets 8–12 reps	RET: 4 LL, 5 UL. Machine- or free-weights	-	Ex 96.8%, sets 96.9%, reps 94.5%	68.2%		11%
	CG	-	Usual care (asked not to initiate any PA). Offered a 1-month exercise program post-IV				-	-	-		27%
Gaskin et al. [34]	IG	Yes	Sup: 2/wk Home: 1/wk	AET: 40–70% APHRM or RPE 8–13 RET/ Flexibility/Balance: NR	AET: 20 min RET: 2 sets 8–12 reps, progressing; Flexibility/Balance: NR (50 min total)	AET: various modes. RET: 2–4 compound exercises for UL, LL & core using theraband, machine and free weights. Flexibility (major muscle groups) and balance	-	NR	85% attended min. 75% session^b^	12 weeks	20%
	CG	-	Usual care (typically involving verbal standard PA advice)				-	-	-		20%
Kampshoff et al. [35]	IG1	Yes	2/wk	LMI: AET = Interval alternating 45/30% MSEC RET = 40–55% 1RM	AET: 2 × 8 min intervals (wks 1–4). Addition of 3 × 5 min aerobic exercise at constant workload (wks 5–12) RET = 2 sets, 10 reps	AET: cycle ergometer, treadmill RET = Body weight, free weight, and machine exercises	3 "booster" sessions provided	NR	91%	12 weeks	20%
	IG2	Yes	2/wk	HI: AET = Interval alternating 65/30% MSEC RET = 70–85% 1RM	AET: 2 × 8 min intervals (wks 1–4). Addition of 3 × 5 min aerobic exercise at constant workload (wks 5–12) RET = 2 sets, 10 reps	AET: cycle ergometer, treadmill. RET = Body weight, free weight and machine exercises	3 "booster" sessions provided	NR	84%		13%
	CG	-	Waitlist control (had completed intervention at 64-week follow-up)				-	-	-		-
Kong et al. [36]	IG1	No	5/wk	NR	If < 150minPA/wk: 3000 steps If ≥ 150minPA/wk: additional 1000–3000 steps	Walking	WAT, counselling, booklet	Mean 11,593 steps/d	NR	5 weeks	28%
	IG2	No	5/wk	NR	If < 150minPA/wk: 30 min If ≥ 150minPA/wk: additional 10-30 min	Walking	Counselling, booklet	NR	NR		17%
Møller et al. [37]	IG1	Yes	Part 1 (wk 1–6): 3/wk training + Part 2 (week 7–12): NR	Part 1: AET: intervals 70-250W (85–95%HR_max_) RET: 5-8RM Part 2: 21–27 MET/wk	AET: 60-90 min/wk RET: 3 sets (Part 1: 9 h/wk total) Part 2: 6 h/wk total	Part 1: AET on stationary bike + RET on machines (UL, LL, core) Part 2: Sports floorball games, dance, & circuit training	Part 1: 1/wk restorative session	NR	70.2%	12 weeks	17%
	IG2	No	5/wk	Low-moderate	30 min/d or 7 500steps/d	Aerobic walking	-	NR	NR		24%
Mutrie et al. [38]& Mutrie et al. [39]	IG	Yes	Sup: 2/wk Home: 1/wk	Moderate (50–75% APHRM)	45 min total	AET, RET or tailored circuit training	Group discussion post-ex	NR	NR	12 weeks	21 months 43% 63 months 56% 9 months 19%
	CG	-	Standard PA recommendations				-	-	-		21 months 45% 63 months 58% 9 months 7%
Penttinen et al. [40]& Vehmanen et al. [46]	IG	Yes	Sup: 1/wk Home: 2–6/wk	Sup: week 1–6 RPE 11–13, progressing to RPE 14–16 Home: RPE 14–16	60 min total	Sup: step aerobic class or circuit training class (alternated weekly) Home: walking, Nordic walking or AET + 96 jumps and leaps	-	NR	All: 3.8/wk^c^, PreM: 3.3/wk, PostM: 4.3/wk^d^	12 months	22%^e^ 17%^f^
	CG	-	Maintain usual PA levels				-	-	-		23%^e^ 17%^f^
Pinto et al. [41]	IG	No	≥ 2/wk—≥ 5/wk	Moderate (55–65% estimated HR_max_)	≥ 10 min—30 min	AET: Brisk walking, biking, or swimming	HCP advice, counselling, printed materials	NR	NR	12 weeks	21%
	CG	-	HCP advice (standard PA recommendations) + contact control				-	-	-		9%
Pinto et al. [42]	IG	No	≥ 2/wk—≥ 5/wk	Moderate (64–76% estimated HR_max_)	≥ 10 min—30 min	Brisk walking, biking or use of home exercise equipment	Counselling, printed materials	NR	NR	12 weeks	5%
	CG	-	Contact control + printed materials				-	-	-		12%
Salerno et al. [43]	IG	No	3/wk	RPE 10–12 (wk 1–2/month), RPE 13–15 (wk 2–4/month)	1–2 sets 8–10 reps (wk 1–2/month), 2 sets 10–12 reps (wk 2–4/month)	RET (body weight and resistance band) + balance and flexibility exercises	Support calls	NR	d/month M1 12, M2 8.7, M3 8.1, M4 7, M5 5.6, M6 5.5	6 months	27%
	CG	-	Standard PA recommendations + attention control calls				-	-	-		38%
SantaMina et al. [44]	IG1	No	3–5/wk	60–80%HRR	30-60 min	AET: walking or individual choice	-	NR	NR	6 months	41%
	IG2	No	3–5/wk	60–80%1RM	30-60 min	RET: resistance bands &ball stability exercises	-	NR	NR		65%
Schmidt et al. [25]	IG	Yes	2/wk	60–80% 1RM	3 sets 8-12reps	RET: 8 machine-based resistance exercises	-	NR	75%^d^	12 weeks	Total 13%
	CG	-	Muscle relaxation sessions. 12wk RET offered post-IV (25% attendance)				-	-	-		
Schmitz et al. [45]	IG	Yes	2/wk	NR	3 sets, 10 reps 90 min total	RET: UL and LL (free and machine weight)	-	NR	NR	13 weeks	14%
	CG	-	Maintain usual PA levels				-	-	-		12%
Witlox et al. [47]	IG	Yes	Sup: 2/wk Home: 3/wk	Sup: AET: 3 × 2 min intervals increasing to 2 × 7 min at or below ventilatory threshold, RET: 2 × 10 reps 65%1RM increasing to 1 × 10reps 75%1RM and 1 × 20reps 45%1RM (60 min total) Home: moderate 30 min		Sup: AET: NR RET: UL, LL, core Home: AET	-	NR	NR	18 weeks	41%
	CG	-	Usual care (maintain habitual PA pattern)				-	-	-		51%

*G* group, *IG* intervention group, *CG* control group, *sup* supervised, *adh* adherence to the intervention, *att* attendance to the intervention, *wk* week, *MET* metabolic equivalent, *h* hour, *d* day, *min* minutes, *M* month, *AET* aerobic exercise training, *RET* resistance exercise training, *HIIT* high intensity interval training, *PreM* premenopausal, *PostM* postmenopausal, *RPE* rating of perceived exertion, *HRR* heart rate reserve, *HRmax* heart rate maximum, *PPO* peak power output, *VT* ventilatory threshold, *MSEC* maximum short exercise capacity, *RM* repetition maximum, *reps* repetitions, *PA* physical activity, *ex* exercise, *UL* upper limb, *LL* lower limb, *IV* intervention, *NR* not reported, *WAT* wearable activity tracker. ^a^At follow-up timepoint, ^b^data from Livingston et al.^44^, ^c^data from Saarto et al. [43], ^d^data reported as median, ^e^dropout data for the participants reported in Penttinen et al. [35], ^f^dropout data for the participants reported in Vehmanen et al. [41]

### Behaviour change theoretical frameworks

Behaviour change theoretical frameworks were reported as informing the intervention in seven of the reviewed trials [33, 34, 36, 41–43, 47]. Three trials [34, 43, 47] framed their intervention using Bandura’s Social Cognitive Theory [48], one trial [33] used the Theory of Planned Behaviour [49], one trial [36] used the Transtheoretical Model [50], and two trials [41, 42] used both the Transtheoretical Model and Bandura’s Social Cognitive Theory.

### Behaviour change techniques

All BCTs identified, including BCT cluster, number, and label, in the included interventions are reported in Table 3. Overall, 24 interventions were analysed for BCTs from the 18 trials. There were six trials [24, 31, 32, 35–37, 44] that included two intervention groups, four [24, 31, 32, 35, 44] of these trials used the same BCTs, and two [36, 37] trials used different BCTs in the intervention groups. Of the 93 BCTs, 27 were coded at least once in the semi-final and final coding. The BCTs coded represented 13 of the 16 BCT clusters. For the individual BCTs based on the semi-final coding, PABAK ranged from 0.67 (BCT 5.3 Information about social and environmental consequences) to 1.0 (mean = 0.94). For the individual interventions, PABAK ranged from 0.91 to 1.0 (mean = 0.98) (see Table 3). Overall, substantial agreement was reached.

**Table 3:** Behaviour change techniques identified in interventions

BCT #	BCT clusters and labels						Arrieta [29]	Baumann [30]	Bolam [31] & Mijwel [24]		Courneya [33]	Courneya [32]		Gaskin [34]	Kampshoff [35]	
							IG	IG^a^	IG1	IG2	IG^a^	IG1	IG2	IG	IG1	IG2
1.0	Goals and planning															
1.1	Goal setting (behaviour)						X		X	X	X	X	X	X	X	X
1.2	Problem solving													X	X	X
1.3	Goal setting (outcome)							X						X		
1.4	Action planning						X		X	X	X	X	X		X	X
1.5	Review behaviour goal															
1.6	Discrepancy between current behaviour and goal															
1.7	Review of outcome goal(s)															
1.9	Commitment															
2.0	Feedback and monitoring															
2.2	Feedback on behaviour						X									
2.3	Self-monitoring of behaviour															
2.7	Feedback on outcome(s) of behaviour															
3.0	Social support															
3.1	Social support (unspecified)						X	X	X	X	X	X	X	X	X	X
3.2	Social support (practical)										X					
4.0	Shaping knowledge															
4.1	Instruction on how to perform the behaviour						X		X	X				X	X	X
5.0	Natural consequences															
5.1	Information about health consequences															
5.3	Information about social & environmental consequences															
6.0	Comparison of behaviour															
6.1	Demonstration of the behaviour								X	X				X		
7.0	Associations															
7.1	Prompts/ cues						X									
7.3	Reduce prompts/cues						X									
8.0	Repetition and substitution															
8.1	Behaviour practice/ rehearsal														X	X
8.6	Generalisation of target behaviour													X	X	X
8.7	Graded tasks															
9.0	Comparison of outcomes															
9.1	Credible source						X							X	X	X
10.0	Reward and threat															
10.4	Social reward										X					
12.0	Antecedents															
12.5	Adding objects to the environment															
13.0	Identity															
13.2	Framing/reframing															
15.0	Self-belief															
15.1	Verbal persuasion about capability															
Sum							8	2	5	5	5	3	3	8	8	8
PABAK							1	1	1	1	1	1	1	0.98	1	1
BCT #	Kong [36]		Møller [37]		Mutrie [38, 39]	Penttinen [40]& Vehmanen [46]	Pinto [41]	Pinto [42]	Salerno [43]	SantaMina [44]		Schmidt [25]	Schmitz [45]	Witlox [47]	Sum	PABAK
	IG1	IG1	IG1^a^	IG2	IG^a^	IG	IG^b^	IG^b^	IG	IG1^b^	IG2	IG	IG	IG^a^		
1.0																
1.1	X	X	X	X	X	X	X	X	X	X	X	X	X	X	23	1
1.2	X	X	X	X			X	X		X	X				11	1
1.3	X	X	X	X											6	0.91
1.4			X	X	X		X	X	X	X	X	X	X	X	19	1
1.5	X	X					X							X	4	1
1.6													X		1	0.92
1.7	X	X													2	0.83
1.9	X	X													2	1
2.0																
2.2	X	X		X			X		X					X	7	1
2.3		X					X	X	X	X	X			X	7	1
2.7								X						X	2	0.92
3.0																
3.1	X	X	X	X	X	X	X	X	X	X	X	X	X	X	24	0.92
3.2															1	0.92
4.0																
4.1			X		X	X	X	X	X	X	X	X	X	X	17	1
5.0																
5.1					X										1	0.83
5.3			X	X											2	0.67
6.0																
6.1						X			X	X	X		X		8	1
7.0																
7.1							X	X	X	X	X				6	1
7.3							X	X	X						4	1
8.0																
8.1					X	X				X	X				6	1
8.6					X	X								X	6	1
8.7	X	X					X	X		X	X				6	0.92
9.0																
9.1					X										5	1
10.0																
10.4								X							2	1
12.0																
12.5		X		X			X	X	X						5	0.83
13.0																
13.2			X	X											2	0.83
15.0																
15.1							X	X						X	3	0.92
Sum	9	11	8	9	8	6	13	13	10	10	10	4	6	10		
PABAK	0.91	0.94	0.96	0.94	1	1	1	0.98	0.96	1	1	1	0.98	0.96		

Coding based on final agreed coding. PABAK based on semi-final coding versions of two reviewers. *PABAK* Prevalence- adjusted bias- adjusted kappa, *IG* intervention group, *BCT* Behaviour Change Technique; #, number; X, BCT present. ^a^Intervention group had significant between-group differences compared with a control/ comparison group; ^b^intervention group had a significant within-group difference only

#### Number and frequency of behaviour change techniques

The number of BCTs used per intervention ranged from 2 [30] to 13 [41, 42] (mean 7.6 BCTs, SD 3.02). The most frequently used BCTs were Social support (unspecified) (*n* = 24) [24, 25, 29–47], Goal setting (behaviour) (*n* = 23) [24, 25, 29, 31–47], Action Planning (*n* = 19), [24, 25, 29, 31–33, 35, 37–39, 41–45, 47] and Instruction on how to perform the behaviour (*n* = 17) [24, 25, 29, 31, 34, 35, 37–47].

#### Implementation of behaviour change techniques

##### BCT cluster: 1.0 Goals and planning

The BCT Goal setting (behaviour) was present in interventions where an exercise goal was set as part of the exercise intervention [24, 25, 29, 31–47]. When the exercise behaviour goal defined a specific context, frequency, duration, or intensity of exercise, the BCT Action planning was also coded [24, 25, 29, 31–33, 35, 37–39, 41–45, 47]. Baumann et al. [30] was the only intervention included in the review where Goal setting (behaviour) was not reported, however the BCT Goal setting (outcome) was reported for the presence of an outcome goal (MET-h/week) of achieving the exercise behaviour.

Goal setting (outcome) was present in five [34, 36, 37] additional interventions, where it was coded for the presence of an outcome goal (e.g. MET-h/week) of achieving the exercise behaviour [36, 37] or the presence of exercise guidelines set as a goal of the intervention [34]. The BCT Problem solving was implemented through counselling or discussion with the participant about identifying and overcoming barriers to physical activity in seven trials [34–37, 41, 42, 44] comprising 11 interventions. The BCT Review behaviour goal was present in three trials [36, 41, 47], comprising four interventions, where the participants’ physical activity goals were reviewed and modified where necessary. Schmitz et al. [45] was the only trial to implement the BCT Discrepancy between current behaviour and goal by telephoning participants who missed exercise sessions. The BCTs Review of outcome goal(s) and Commitment were only implemented by Kong et al. [36] in both intervention groups of the trial, by reviewing and modifying the outcome goal and making goal decisions with patient agreement, respectively, in both intervention groups.

##### BCT cluster: 2.0 Feedback and monitoring

From the BCT cluster Feedback and monitoring, the BCT Feedback on behaviour was used in six trials consisting of seven interventions [29, 36, 37, 41, 43, 47]. Feedback on behaviour was implemented through instructors providing feedback on physical activity performed [29, 47], counselling session involving the evaluation of and feedback on physical activity levels [36], a computer-based programme where participants can visualise their performance of physical activity [37], or participants received a letter of feedback on their physical activity progress [41, 43]. Self-monitoring of behaviour was implemented in seven interventions [36, 41–44, 47] through a wearable activity tracker [36], participants recording their physical activity (not for outcome purposes) [43, 44, 47], and two interventions noted training participants in techniques of self-monitoring of physical activity [41, 42]. The BCT Feedback on outcome(s) of behaviour were used in two interventions [42, 47], through telephone calls where participants received feedback on their physical activity log [42] and feedback on their obtained results from an exercise professional [47].

##### BCT cluster: 3.0 Social support

The BCT Social support (unspecified) was applied through various methods, including individual or group counselling [29, 35–37, 41, 42], telephone support calls [43–45], and motivation and encouragement from exercise specialists [24, 25, 30–34, 38–40, 46, 47]. Social support (practical) was only implemented in one intervention [33] through phone-calls from staff when participants missed more than one session per week.

##### BCT cluster: 4.0 Shaping knowledge

The BCT Instruction on how to perform the behaviour was coded in 14 trials, comprising 17 interventions, where participants attended supervised sessions and instruction of exercise was specified [24, 25, 31, 34, 35, 37, 45], participants attended an exercise class [38, 39, 47], verbal instruction was provided on how to exercise [29, 41, 42, 44], and for a DVD-delivered instructional program [43]. This was the only BCT coded for the cluster Shaping knowledge.

##### BCT cluster: 5.0 Natural consequences

The BCT Information about health consequence was implemented in one intervention [38, 39] with Information about social and environmental consequences implemented in two interventions of the same trial [37]. These BCTs were only coded when there was sufficient detail that information on the respective consequences were provided to participants. For example, ‘discussion of health benefits of exercise’ [38, 39] and ‘information regarding the benefits of physical activity’ [37].

##### BCT cluster: 6.0 Comparison of the behaviour

The BCT Demonstration of the behaviour was reported in eight interventions across six trials [24, 31, 34, 40, 43–46]. Only three interventions across two trials [24, 31, 34] included a supervised intervention and provided sufficient detail in the methods that participants received demonstration of exercise. Demonstration of the behaviour was implemented through group exercise classes in two interventions [40, 45, 46]. Salerno et al. [43] provided DVD led exercise sessions where the exercise leader demonstrated modified and challenging versions of the exercises. Lastly, Santa Mina et al. [44] provided each participant with detailed exercise instructions with demonstration in both intervention groups.

##### BCT cluster: 7.0 Associations

Two of the possible eight BCTs from the cluster Associations were coded in the included interventions. The BCTs Prompts/cues and Reduce prompts/cues were implemented together in four interventions [29, 41–43], and Prompts/cues in one additional trial containing two interventions [44]. These BCTs were implemented in a similar manner across interventions, where participants received regular phone calls to prompt physical activity behaviour (Prompts/cues), with the frequency of calls reducing throughout the intervention (Reduce prompts/cues).

##### BCT cluster: 8.0 Repetition and substitution

The BCTs Behaviour practice/rehearsal [35, 38–40, 44, 46] and Generalisation of target behaviour [34, 35, 38–40, 46, 47] were coded in six interventions, with four [35, 38–40, 46] of these interventions containing both BCTs. Behavioural practice/rehearsal was only coded where booster sessions were provided [35, 44] or where the participants attended exercise classes (as per the BCT taxonomy) [38–40, 46]. Generalisation of target behaviour was coded when participants were advised to perform physical activity that was performed in a supervised setting, and also at home [35, 38–40, 46, 47].

In the final coding, Graded tasks was coded in six intervention groups [36, 41, 42, 44], from four trials. The BCT Graded tasks was only coded for interventions that provided adequate description to indicate that exercise progression was also being used as a method of behaviour change and not solely as an exercise prescription principle.

##### BCT cluster: 9.0 Comparison of outcomes

From the BCT cluster Comparison of outcomes, the BCT Credible source was the only BCT coded. The BCT was only coded where the methods provided sufficient detail that the credible source (i.e. a health or exercise professional, e.g. exercise physiologist) specifically communicated in favour of or against the behaviour. Thus, five interventions [29, 34, 35, 38, 39] were coded for the BCT Credible source.

##### BCT cluster: 10.0 Reward and Threat

The BCT Social reward was the only BCT implemented from the cluster Reward and Threat. Two interventions [33, 42] reportedly implemented Social reward through positive reinforcement from trial staff for the performance of physical activity.

##### BCT cluster: 12.0 Antecedents

The BCT Adding objects to the environment was included in five interventions [36, 37, 41–43]. This BCT was implemented by two methods: providing participants with a wearable activity tracker (pedometer or Fitbit) [36, 37, 41, 42] or exercise equipment [43].

##### BCT cluster: 13.0 Identity

Møller et al. [37] implemented the BCT Framing/reframing in both trial interventions through counselling sessions by switching the focus of physical activity on improving cancer-related side effects.

##### BCT cluster: 15.0 Self-belief

Of the four possible BCTs in the cluster Self-belief, Verbal persuasion about capability was the only BCT coded, and was implemented in three interventions [41, 42, 47]. In two interventions [41, 42], Verbal persuasion about capability was implemented through counselling that included building confidence in becoming/staying active. Witlox et al. [47] included verbal persuasion as a method to increase self-efficacy.

### Maintenance of physical activity at follow-up

Of the 21 articles included in this review, five (23.81%) articles [30, 33, 37, 38, 47] reported significant between-group differences favouring an intervention group ≥ 6 months following the end of a structured exercise intervention. The remaining 16 articles (76.19%) reported no significant between-group differences (Table 1).

#### Between- and within-group differences

Of the 21 articles included in this review, four articles (19%) [30, 33, 38, 47] reported significant between-group differences in physical activity ≥ 6 months following the completion of an exercise intervention, favouring the intervention compared with a control group. In the article by Baumann et al. [30], between- (mean difference (MD) =  + 1294MET-min/week, *p* = 0.005) and within-group (MD =  + 4.13 h/week, *p* = 0.001) improvements in total physical activity levels were observed 23 months post-intervention completion in the exercise group. The authors also reported significant between-group differences favouring the exercise intervention at 11 months follow-up (MD =  + 1422 MET-min/week, *p* = 0.005), but not at 7 months (MD =  + 960MET-min/week, *p* = 0.02), or 17 months follow-up (MD =  + 595MET-min/week, *p* > 0.05). In Witlox et al. [47], total physical activity levels were significantly higher in the intervention group 3.6 years post-intervention completion (MD =  + 141.46 min/week; ES = 0.22; *p* < 0.05) compared to the control group. The authors noted a significant increase in sport and leisure-related physical activity levels (MD =  + 85.18 min/week, *p* < 0.05), but not total physical activity levels (MD =  + 43.22 min/week, *p* > 0.05) in the intervention group from baseline to 3.6 years follow-up [47]. No significant within-group changes in total or sport and leisure-related physical activity levels were observed at follow-up in the control group (MD = -143.77 min/week and + 54.67 min per week, respectively; all *p* > 0.05) [47]. Courneya et al. [33] reported a larger number of participants in the intervention group engaging in regular physical activity 6 months following the end of the intervention (MD =  + 23.6%; *p* = 0.017) compared to the control group. Mutrie et al. reported on follow-up outcomes of the same trial in two articles at 6 months [39], and 18 months and 5 years [38] post-intervention. Whilst there were no significant between-group differences at 6 (*p* = 0.23) [39] or 18 months follow-up (*p* = 0.22) [38], at 5 years follow-up, a significant effect estimate (*p* = 0.008) was observed favouring the intervention group compared with the control group [38].

Møller et al. [37] compared two different interventions: a supervised multi-modal exercise intervention versus an unsupervised aerobic walking program. Whilst the percentage of participants performing 150 min of moderate-to-vigorous physical activity per week was not different between groups 6 months post-intervention (percentage not reported, *p* = 0.1270), a higher percentage of the multi-modal exercise group performed two 20-min sessions of high intensity physical activity per week (percentage not reported, *p* = 0.0408). Moller et al. [37] also noted a significant within-group increase from screening, baseline to 6 months post-intervention in the percentage of participants in both groups performing > 150 min per week of moderate-to-vigorous physical activity (percentages not reported, *p* < 0.0001 and *p* < 0.0039, respectively) and > 2 × 20 min/week sessions of high intensity physical activity (percentages not reported, *p* < 0.0001 and *p* < 0.0004, respectively).

Of the 21 articles included in this review, three articles [41, 42, 44] reported significant within-group differences in an intervention group, with no significant between-group differences. Pinto et al. [42] reported a significant improvement in physical activity levels 9 months post-intervention cessation in the intervention group (MD =  + 116 min/ week, *p* < 0.05), but not in the control group (MD =  + 58 min/ week, *p* value not reported). A second article by Pinto et al. [41] reported a significant increase in physical activity at 9 months follow-up in the intervention group (MD =  + 1.16 min/ week, *p* < 0.05) and a significant decrease in the control group (MD = -11.19 min/week, *p* < 0.05). Santa Mina et al. [44] reported a significant increase in physical activity from baseline to the 6 months follow-up (MD =  + 13.68 MET-h/week, *p* ≤ 0.06) in the AET group. However, there was no significant changes in physical activity levels in the RET group (MD =  + 2.98MET-h/week, *p* > 0.05) [44]. None of the included articles reported significant increases in physical activity levels within the control groups.

#### BCTs and maintenance of physical activity at follow-up

The five trials [30, 33, 37, 38, 47] that observed a between-group difference in physical activity at follow-up favouring the intervention group included a mean of 7 BCTs (range 2–10). Those studies that observed no between-group differences [24, 25, 29, 31, 32, 34–36, 39–46] in physical activity included 8 BCTs (range 3–13). Of the five [30, 33, 37, 38, 47] intervention groups that reported significant differences in their favour, Social support (unspecified) was the only BCT present in all five groups. Though Social support (unspecified) was also present in all remaining exercise interventions that did not report significant between-group differences. The BCTs Goal setting (behaviour) and Action Planning were present in four [33, 37, 38, 47] of the five (80%) interventions reporting significant between-group differences in their favour. Goal setting (behaviour) and Action Planning were also frequently used across all interventions, with 96% and 79%, respectively, of the 24 interventions including these BCTs. Instruction on how to perform the behaviour was the next most frequently used BCT within the interventions reporting significant between-group differences, with three (60%) [37, 38, 47] of the five interventions utilising this BCT. Two (40%) of the interventions that reported significant differences in their favour, implemented Goal setting (outcome) and Generalisation of target behaviour. The remaining 14 BCTs utilised across the interventions that reported significant between-group differences in their favour were used in only one of the five interventions.

The three trials [41, 42, 44] that observed a within-group difference in physical activity at follow-up in an intervention group included a mean of 12 BCTs (range 10–13). There were eight BCTs present in all three interventions with significant within-group differences; these included Goal setting (behaviour), Problem Solving, Action Planning, Self-monitoring of behaviour, Social support (unspecified), Instruction on how to perform the behaviour, Prompts/cues, and Graded tasks. Further, three BCTs (Reduce prompts/cues, Adding objects to the environment, and Verbal persuasion about capability) were present collectively in two (67%) [41, 42] of the interventions reporting significant within-group differences. The remaining six BCTs that were implemented across interventions with significant within-group differences were used in only one of the three interventions.

## Discussion

This systematic review aimed to determine the long-term (≥ 6 months) maintenance of physical activity following an exercise intervention in individuals with a histologically confirmed diagnosis of cancer. A total of 21 articles were identified, consisting of 18 randomised controlled trials. There was high heterogeneity in trial design, intervention characteristics, length of follow-up, and BCTs used in the included trials. Based on the available evidence, long-term physical activity following an exercise intervention appears to be poorly maintained in people living with and beyond cancer. There appears to be no clear implementation of a behaviour change theory to an intervention, BCT, or combination of BCTs, that contributes to enhanced long-term maintenance of physical activity.

Of the 21 articles, five (24%) [30, 33, 37, 38, 47] found significant between-group differences in physical activity ≥ 6 months follow-up favouring the intervention compared to a control/comparison group. This finding suggests that long-term (≥ 6 months) physical activity is poorly maintained following an exercise intervention in people with cancer. This result is contrary to Grimmett et al. [14] who reported a small positive effect in interventions compared with a control group on physical activity behaviour at ≥ 3 months follow-up. However, their review included multimodal interventions and only articles that reported physical activity in moderate-to-vigorous minutes per week were included in the review and subsequently, the meta-analysis [14]. Further, our results are consistent previous systematic reviews that assessed change in physical activity in men with prostate cancer [16] and found in only two (17%) of the included articles, physical activity was maintained at 3–6 months follow-up, with one of those trials also demonstrating maintenance > 6 months; and in people living with and beyond breast cancer [15] where only 30% maintained physical activity ≥ 3 months post-intervention. A low proportion of trials include a follow-up ≥ 6 months post-intervention, as indicated by the extensive number of trials assessing exercise interventions in people living with and beyond cancer compared with the 18 trials included in this review. Future research needs to focus on long-term physical activity maintenance and include an assessment of physical activity levels ≥ 6 months post-intervention completion to build the understanding of long-term physical activity maintenance in people with cancer.

Only two [33, 47] of the five articles that found between-group differences identified a theoretical basis for the intervention, the Theory of Planned Behaviour and Bandura’s Social Cognitive Theory. A further five [34, 36, 41–43] articles that found no difference between groups at follow-up used a theoretical basis, including the Theory of Planned Behaviour, Bandura’s Social Cognitive Theory, and the Transtheoretical Model, for the intervention. This is consistent with previous systematic reviews that reported no trend on the use of behaviour change theories to promote physical activity in people with cancer [12, 51]. This suggests that the use of a behaviour change theory alone is not enough to promote long-term maintenance of physical activity in people with cancer. Previous work has identified the complexity of behaviour change maintenance and the lack of theoretical elaboration on behavioural maintenance after the initial stages of change [18], which may explain the dissociation between theories and practice. Further, a probable cause is that the evidence and specifically the application of behaviour change theories to practice is diverse and not clearly articulated. Trials are rarely explicit about the use of behaviour change theories or when a theory is included, there is inadequate explanation of how the intervention theories are applied in practice [12, 51, 52].

Behaviour change techniques were identified in all articles included in the review [24, 25, 29–47] with the aim to identify patterns in the active intervention components that promote long-term physical activity behaviour change in people with cancer. The total number of BCTs present were similar for interventions that observed significant between-group differences in physical activity levels at follow-up and those that found no effect. Of note, Baumann et al. [30] only implemented two BCTs and noted significant between- and within-group differences in physical activity levels at follow-up favouring the intervention group. Therefore, the total number of BCTs alone does not appear to impact the maintenance of physical activity levels ≥ 6 months following completion of an exercise intervention. This is consistent with previous reviews reporting no effect of total number of BCTs used to increase or maintain physical activity levels [10, 53]. Rather than the number of BCTs, it is likely that the application and combinations of BCTs influence changes in physical activity behaviour. The clinical and interpersonal skills employed in delivering an intervention are likely to play a crucial role in maintain behaviour change [54]. Thus, whilst it’s important to recognise which BCTs are being utilised in interventions, more in-depth analysis is necessary to comprehend how they are being implemented and what ultimately leads to successful behaviour change.

Of the five articles [30, 33, 37, 38, 47] reporting significant between-group differences, there was overlap in the BCT clusters 1: Goals and planning, and 3: Social support, with all five articles containing at least one BCT from these clusters. The most prevalent BCTs within these clusters were Goal setting (behaviour), Social support (unspecified), and Action planning. Baumann et al. [30] was the only article with significant between-group differences not to include the BCT Goal setting (behaviour), though it did include the BCT Goal setting (outcome). Goal setting has consistently been recognised as an important technique for behaviour change [55, 56], including for physical activity [57]. Social support (unspecified) and Action planning have also been identified as important for short-term (≥ 3 months) maintenance of physical activity following an exercise or multimodal health intervention [14]. Future exercise interventions for people living with and beyond cancer should include goal setting, social support, and action planning to enhance the likelihood of participants maintaining their physical activity levels long-term following completion of the intervention. However, the BCTs Goal setting (behaviour), Action planning, and Social support (unspecified) were also present in many interventions that did not observe significant between-group differences in physical activity at follow-up. Whether these BCTs are necessary for change but need to be used in combination with other BCTs is unclear. The BCTs Goal setting (behaviour or outcome) and Action planning were operationalised through setting a goal as part of the intervention, and through planning the performance of the behaviour, respectively. Social support was operationalised through support directed at physical activity from peers or staff, delivered by face-to-face or telephone calls. Including BCTs from the clusters Goals and planning and Social support as they have previously been operationalised appears necessary but not sufficient to promote physical activity long-term following an exercise intervention.

With the substantial overlap of BCTs used among interventions, it is difficult to determine the efficacy of individual BCTs to promote physical activity behaviour. Further, a regression analyses was not performed because of the heterogeneity of the included trials. Of the interventions that reported significant between-group differences in their favour, Instruction on how to perform the behaviour was used in three (60%) interventions, Goal setting (outcome) and Generalisation of target behaviour were used in two interventions (40%), and a further 14 individual BCTs were used only once (20%). By comparison, Instruction on how to perform the behaviour was used in 14 (74%) interventions, Goal setting (outcome) in four (21%) interventions and Generalisation of target behaviour in four (21%) interventions that reported no significant between-group differences. The use of multiple BCTs within the majority of interventions limits the ability to detect the isolated benefit of any individual BCT. Future research directly comparing the efficacy of different BCTs would provide greater insight into which BCTs would be most beneficial to encourage long-term maintenance of physical activity following an exercise intervention for people with cancer.

Supervision also appears to be a necessary, but insufficient in isolation, component of an exercise intervention to maintain long-term physical activity levels. Excluding one article that did not specify supervision status [30], all articles included in this review that observed significant between-group differences favouring the intervention in long-term physical activity provided supervised intervention elements. Two previous systematic reviews concluded that supervised exercise programs are superior to unsupervised programs for increasing physical activity in oncological populations [58, 59]. One could argue that it is not the supervision per se that may be important for physical activity maintenance, but rather that supervised interventions can include additional BCTs and be a method for facilitating BCTs compared to unsupervised interventions. For example, supervised interventions can include the BCTs Social support (unspecified), Instruction on how to perform a behaviour, and Demonstration of the behaviour. It is also probable that BCTs were implemented where supervision by an exercise professional was used but was not sufficiently detailed in the methods to code. For example, information about the consequences of physical activity (health or other) (BCT cluster 5.0), or positive reinforcement as a form of reward (BCT cluster 10.0) could have been provided to participants during supervised sessions but not included in the methodology. Many of the BCTs implemented in supervised interventions were also implemented in unsupervised interventions through different methods. For example, all unsupervised interventions [29, 36, 41–44] implemented Social support (unspecified) through telephone calls. Further, Salerno et al. [43] implemented Instruction on how to perform a behaviour and Demonstration of the behaviour through DVD-led exercise sessions. Despite similar BCTs between supervised and unsupervised interventions, the incorporation of supervision within exercise interventions may provide value through the inherent factor of supervision (e.g. the personal connection and individualised communication) or the operationalisation of BCTs within a supervised setting. Alternatively, implementing another model after supervision ends (e.g. peer support) may be useful to promote long-term motivation and relapse prevention.

A key limitation of the review by Grimmett et al. [14] was the exclusion of articles that targeted RET. In the present review, four articles [25, 32, 44, 45] included a RET-only intervention group, but none reported significant between- or within-group differences at follow-up ≥ 6 months post-intervention. Santa Mina et al. [44] compared a home-based AET versus a home-based RET group, implementing the same BCTs in both groups. The AET group performed a significantly greater volume of physical activity at follow-up, whereas the RET did not significantly increase physical activity levels at follow-up; though there was no significant differences between-groups [44]. It was suggested that AET mode (e.g. walking) is more familiar and thus more easily reproducible in the absence of instruction or demonstration compared to RET. Whilst the BCTs on Instruction how to perform the behaviour and Demonstration of the behaviour were implemented in all RET interventions, the dosage and/or frequency may not have been sufficient to elicit long-term behaviour change. A limitation of RET interventions is the methods used to measure physical activity are biased towards AET, and may not appropriately capture levels of RET. Self-report measures often solely use examples of AET modalities [60, 61] and device-based measures do not provide accurate data on RET [62]. Therefore, whilst RET interventions do not appear to induce long-term maintenance of physical activity following completion of the intervention, better tools to monitor RET such as those proposed by Fairman et al. [63, 64] are recommended in future interventions to identify potential changes more accurately in physical activity behaviour in oncology populations.

This study highlights the importance of integrating evidence-based exercise prescription with behavioural science for physical activity maintenance. To continue to grow evidence in this area, researchers should explore the use of BCTs and their combinations to enhance physical activity interventions, and clearly report the BCTs used and how they have been implemented. Researchers and clinicians should collaborate to optimise the use of BCTs in a clinical environment. For clinicians, evidence-based practice should not only be applied to prescribing exercise, but also to behaviour change strategies, such as BCTs. The BCTs Social support, Goal setting (behaviour), and Action planning were present in interventions that led to physical activity maintenance, and therefore it is recommended that clinicians incorporate these techniques in their clinical practice. Although integrating behavioural science into physical activity interventions is complex, it is crucial for researchers and clinicians to incorporate these methods to enhance effectiveness of interventions, which can lead to improved physical activity maintenance and associated benefits for individuals. Further, utilising existing resources (e.g. The Behaviour Change Wheel [65] and works by O’Cathain et al. [66]) can guide researchers, clinicians and also policy makers on intervention development.

### Limitations and future directions

This systematic review has several limitations worthy of comment. Firstly, the majority of participants included in this review were female and diagnosed with breast cancer. Therefore, the findings of this review should be interpreted with caution, especially when applying to other oncological populations. The methods chosen to measure physical levels in the included articles are another notable limitation. Of the 21 articles included in this review, 17 (81%) utilised self-report questionnaires to measure physical activity, which tend to over-estimate physical activity levels compared to device-based methods [67]. Whilst more objective measures of physical activity may be considered an appropriate response to this concern, device-based measures such as accelerometers present their own constraints. Most salient is their limited comprehensiveness in detecting all physical activity [62]; accelerometers cannot provide accurate data on cycling, RET, balance, or aquatic-based activities, modes of exercise frequently prescribed within exercise oncology interventions. Long-term (≥ 6 months) follow-up data post-intervention is lacking within the exercise oncology literature. Inadequate follow-up was the primary reason for trial exclusion in this review. Further, only 28% (5/18) of the included trials reported physical activity as the primary outcome measure. Despite physical activity not being the primary outcome in these trials, an exercise intervention was used to facilitate changes in the primary outcome. Future exercise interventions need to include long-term (≥ 6 months) follow-up timepoints, to enhance understanding of the components of interventions, including BCTs, that may promote long-term maintenance of physical activity.

Exercise interventions seldom describe BCTs with sufficient detail to appropriately interpret the study findings [68]. Therefore, it is possible that BCTs were implemented in the included articles but not adequately coded. Where present, published protocol papers describing methods were included in this review. The assumption was made that if a BCT was present in a protocol paper, it was implemented in the trial with the published results. In addition to the presence of BCTs, the quality and delivery of the BCT can influence the effective implementation and contribution of a BCT [69]; however, detailed description of implementation techniques is rarely reported in intervention methodology. A similar limitation exists in the use of behaviour change theories in interventions. The application of behaviour change theories to practice is diverse and not clearly articulated. Trials are rarely explicit about the use of behaviour change theories, or when a theory is included, and inadequately explain how the intervention theories are applied and evaluated in practice [12, 51, 52].

Less than 30% (27/93) of the BCTs available in the BCT Taxonomy (version 1) were coded in the included articles. This percentage is similar to previous reviews that have coded 23–40% of the possible BCTs in exercise oncology trials [10, 14, 53]. Further, with the substantial overlap of BCTs used among the included interventions, there is limited diversity in the exercise oncology literature of BCT use. Although some BCTs are not suitable to be applied in an intervention aimed at changing physical activity behaviour (e.g. Pharmacological support or Behaviour cost), future interventions should explore many of the underutilised BCTs to determine their effectiveness at increasing long-term physical activity in oncological populations.

Maintenance of behaviour change was defined according to the transtheoretical model of behaviour change [50] as ≥ 6 months follow-up in order to provide a consistent cut-off time-point to examine maintenance in this review. However, there is no consensus regarding the utility of stages in the transtheoretical model, the length of time it may take an individual to reach a particular stage, or how long they may remain in a stage [70]. More contemporary definitions of maintenance reject potentially arbitrary definitions and the distinct separation of maintenance as a stage, and instead suggest maintenance is recognised as a process that involves intentionally changing behaviour and continuously performing it at a greater level of efficiency than before [18, 71]. Future research should consider contemporary descriptions of physical activity maintenance, and investigate BCTs that may be utilised throughout the process of maintenance of behaviour change.

## Conclusion

The findings of this research indicate that the long-term maintenance of physical activity following an exercise intervention for people with cancer is limited and inconclusive. The presence of BCTs was similar across interventions with significant differences in physical activity and interventions with no significant differences. To strengthen understanding of the use of BCTs in the literature, articles should provide precise and detailed explanations of methods used to increase behaviour change, to permit accurate coding or explicitly report the behaviour change techniques used according to standardised coding frameworks [27]. Future interventions should focus on using different BCTs and combinations of BCTs in intervention design to enhance long-term physical activity behaviours in people with cancer.

## Supplementary Information

Below is the link to the electronic supplementary material.

Supplementary file1 (PDF 97 KB)

Supplementary file2 (PDF 29 KB)

## Data Availability

All data are included in the published article and its supplementary files, and the complete datasets are available from the corresponding author on reasonable request.
