# Supplementary material for: Physical activity behaviour change in people living with and beyond cancer following an exercise intervention: a systematic review
Source: J Cancer Surviv. 2023 Apr 19;17(3):569–94. doi: 10.1007/s11764-023-01377-2 (PMC10209249; doi:10.1007/s11764-023-01377-2)
Supplement: Supplementary file 1 — Supplementary file1 (PDF 97 KB) [file 11764_2023_1377_MOESM1_ESM.pdf]

Supplementary File 1: Search strategy

Example search: CINAHL

Search ID#	Search terms	Results
S1	MH Neoplasms	85181
S2	Neoplasm* OR cancer OR carcinoma	673,741
S3	S1 OR S2	673741
S4	MH Exercise	55543
S5	Exercis* OR “Physical activity” OR “Weight training” OR Resistance OR Strength OR Endurance OR Aerobic	439479
S6	S4 OR S5	439479
S7	Program* OR intervention*	946425
S8	S3 AND S6 AND S7	1039
S9	MH Muscle strengthening	12885
S10	MH Physical Activity	44567
S11	MH Resistance Training	6060
S12	S4 OR S5 OR S9 OR S10 OR S11	441491
S13	S3 AND S7 AND S12	1042
