# Supplementary material for: Physical activity behaviour change in people living with and beyond cancer following an exercise intervention: a systematic review
Source: J Cancer Surviv. 2023 Apr 19;17(3):569–94. doi: 10.1007/s11764-023-01377-2 (PMC10209249; doi:10.1007/s11764-023-01377-2)
Supplement: Supplementary file 2 — Supplementary file2 (PDF 29 KB) [file 11764_2023_1377_MOESM2_ESM.pdf]

Supplementary File 2: Intervention details

Exercise intervention

Frequency

Exercise frequency was reported in 17 [24, 25, 29, 31-47] of the 18 trials included in this review. Exercise frequency ranged from two to seven days per week, with aerobic exercise training (AET) interventions [32, 33, 36, 37, 40-42, 44, 46] ranging from two to seven days per week, and resistance exercise training (RET) interventions [25, 32, 44, 45] from three to five days per week. Interventions featuring a combination of AET and RET [35, 38, 39, 47] were prescribed at a frequency of two to five days per week; interventions with >two modes of exercise [29, 34, 37, 43] from two to three days per week, and high intensity interval training (HIIT) [24, 31] at a frequency of two days per week. Baumann et al. [30] prescribed a total volume of exercise, with participants instructed to accumulate 15 metabolic equivalent (MET) hours/week; specific details of frequency, intensity and duration of exercise were not reported.

Intensity

Of the 18 trials, 16 [24, 29-35, 37-47] reported exercise intensity. The AET interventions prescribed moderate-to-vigorous intensity exercise defined as between 60-80% maximum aerobic power, peak power output or heart rate reserve [32, 33, 44], or rating of perceived exertion (RPE 11-13 progressing to 14-16). [40, 46]. All RPE scales in the included articles used a scale of 6-20. Moderate intensity AET was prescribed by two interventions, defined as 55-65% [41], and 64-76% [42] of age-predicted heart rate maximum. The RET interventions that reported intensity [25, 32, 44] were prescribed at 60-80% one repetition maximum (RM). Interventions that prescribed a combination of AET and RET used varied methods of monitoring and reporting intensity. Two of these interventions prescribed global intensity for both AET and RET, with one [29] reporting low-high intensity and focused on avoiding pain and exhaustion, without further specification; and the other [38, 39] reporting prescription at a moderate intensity (50-70% age-predicted heart rate maximum). Møller et al. [37] prescribed AET at 70-250W or 95-95% heart rate maximum, and RET as 3 sets at 5-8RM for part one of the intervention (week 1-6), but only reported weekly metabolic equivalent (21-27) for part two of the intervention (week 7-12). The article by Kampshoff et al. [35] compared a combined aerobic interval training and RET program at a high intensity versus a low-moderate intensity. Aerobic interval training was prescribed using maximum short exercise capacity (65/30% and 45/30%, respectively) and RET was prescribed using 1RM (70-85% and 40-55%, respectively). Witlox et al. [47] prescribed AET as at or below ventilatory threshold and RET as percentage of 1RM (45%-75%). Gaskin et al. [21] prescribed moderate intensity AET through RPE (8-13) and age-predicted heart rate maximum (40-70%) methods; RET intensity was not reported. Salerno et al. [43] prescribed an RPE of 10-12 progressing to 13-15 for a RET and balance intervention. The two intervention groups in both Bolam et al. [31] and Mijwel et al. [24] prescribed HIIT at RPE 16-18, interspersed with active rest at RPE 13-15, AET at RPE 13-15, and RET at 70% 1RM, increasing to 80% across the intervention.

Time

Total exercise duration was reported in nine [24, 31-34, 38-40, 44-47] of the 18 trials, ranging from 15 to 90 minutes (mean= 57 minutes). Interval exercise was utilised in five articles [24, 31, 35, 37, 47] reporting on four trials. One trial did not report the interval duration [37] and the remaining three trials [24, 31, 35, 47] reported total interval training duration ranging from two to eight minutes, with the working duration ranging from 30 seconds to seven minutes and the active recovery duration ranging from 30 seconds to one minute. Witlox et al. did not report the active recovery duration [47]. RET was utilised in 11 articles, [24, 25, 29, 31, 32, 34, 35, 37, 43, 45, 47] of ten trials, where RET was prescribed based on the number of exercises, sets, and repetitions. These ranged from six to nine exercises of one to three sets, and eight to 20 repetitions.

Mode

Exercise interventions involved AET only [32, 33, 36, 37, 40-42, 44, 46], RET only [25, 32, 44, 45], a combination of both AET and RET at various intensities [35, 38, 39, 47], AET, RET, balance and flexibility training [29, 34], RET, balance, and flexibility training [43], AET, RET, floorball games, dance, and circuit

training [37], high intensity interval training of various modes [24, 31], or the participants preferred mode of exercise [30].

Of the 18 trials included, 15 [24, 25, 29, 31-37, 40-46] reported the type of exercise prescribed. Types of AET included cycle ergometry or biking [24, 31-35, 37, 41, 42], walking or jogging (treadmill or outdoors) [24, 31, 32, 34-37, 40-42, 44, 46], elliptical training [24, 31, 32, 34], rowing ergometry [34], swimming [41], or step and circuit classes [37, 40, 46]. Arrieta et al. [29] reported individualised AET, but no mode was specified. Types of RET included bodyweight [35, 43], machine- or free-weights [24, 25, 31, 32, 34, 35, 37, 45], resistance bands [34, 43, 44], and stability balls [44]. All flexibility, balance, and proprioception training were completed using body weight exercises [29, 34, 43]. One article [30] did not prescribe a specific AET or RET, but asked participants to engage in their preferred mode of exercise during the intervention [30]. If the participants could not choose, the researchers assigned the participants a suitable activity (walking, treadmill, ergometer, cycling, machine weights training) based on their physical capabilities [30]. Three articles [38, 39, 47] comprising two trials reported that individualised AET and RET was prescribed, but specific mode was not specified.

Supervision

Supervised interventions were present in 11 trials of the 18 trials included in this review [24, 25, 31-35, 37-40, 45-47], nine were supervised by an exercise specialist, [24, 31, 33-35, 37-40, 45-47] and two provided insufficient detail to determine the supervising provider [25, 32]. In addition to the supervised component, four of these trials also included an unsupervised component [34, 38-40, 46, 47]. Unsupervised interventions were the primary delivery method in six trials [29, 36, 41-44], with four facilitated through telephone advice. The intervention groups in the trial by Santa Mina et al. [44] were primarily unsupervised, with 12 optional supervised sessions in conjunction with a fortnightly telephone call from an Exercise Physiologist. The intervention group in the trial by Salerno et al. [43] received a DVD-delivered exercise intervention. Baumann et al. [30] did not specify whether the intervention was supervised.

Adherence and attendance

Adherence to the exercise prescription in the intervention groups were reported in four trials (22%) [24, 31-33, 36], with inconsistencies in reporting. Two trials [32, 33] reported adherence to the exercise duration and intensity in the AET groups. Adherence to the duration and intensity were 99% and 90.7% [33], 95.6% and 87.2% [32], respectively. Further, Courneya et al. [32] reported adherence to the RET group in terms of the exercises (96.8%), sets (96.9%), and repetitions completed (94.5%). Bolam et al. [31] reported adherence to the exercise intervention based on participants who successfully completed 90% of the exercise sessions according to the prescription (i.e., intensity and duration), divided by the total number of participants in the intervention groups. Adherence was 75% and 83% in the AT-HIIT and RT-HIIT groups, respectively. The same data were used for adherence in the article by Mijwel et al. [24] who reported on the same trial with a different follow-up timeframe. Kong et al [36] reported adherence in only one of the two intervention groups included in the trial, where adherence was reported in the as mean daily step count (11,593) during the intervention.

Attendance to the exercise program in the intervention groups were reported in 10 trials (56%) [24, 25, 29, 31-35, 37, 40, 43, 46], with eight of these trials including a supervised component. Various methods were used to calculate and report attendance to the exercise training sessions. Mean session attendance was the most commonly reported method for supervised sessions (60%) [24, 31-33, 35, 37, 40, 46], with results ranging from 63% [24, 31] to 91% [35]. In the trial by Penttinen et al. [40] and Vehmanen et al. [46] mean attendance to the supervised training sessions was 62%, and of the participants that returned their training diaries (88%) mean home training sessions were 3 sessions per week, resulting in a median of 3.8 sessions per week total [68]. Schmidt et al. [25] reported attendance as a median value (75%). Attendance data for the trial by Gaskin et al. [34] was reported by Livingston et al. [69], who reported that 85% of participants attended at least 75% of the supervised sessions.

There were two trials that reported attendance to an unsupervised exercise program. Arrieta et al. [29] reported that 70.1% of physical activity advice was declared as effectively performed by participants with a complete follow-up, no further details were reported. Salerno et al. [43] reported the mean exercise days per month (7.82 sessions per month) over the 6-month intervention, with months ranging from 5.5 sessions per month to 12 sessions per month.

Control/ Comparison group

Of the 18 trials included in this review, there were 15 control groups and 24 intervention groups (see Table 2). There were 12 trials with a control group and an intervention group [25, 29, 30, 33, 34, 38-43, 45-47] three trials with two intervention groups [36, 37, 44], and three trials with a control group and two intervention groups [24, 31, 32, 35]. Of the 15 control groups, five received usual care where they were asked to maintain their habitual physical activity levels [32, 33, 40, 45-47], five received usual care where they were provided with standard physical activity recommendations [24, 29-31, 34, 38, 39], three articles used a contact control in addition to standard physical activity recommendations [41-43], one trial used muscle relaxation activities [25], and one trial had a wait-list control group.

Follow-up period

The follow-up periods in the articles included in this review ranged from six months [32, 33, 36, 37, 39, 43, 44] (as determined by the inclusion criteria) to five years [38] (see Table 1).

During the follow-up period, three articles [32, 36, 43] withdrew all contact and support, and six articles [29, 33, 40, 44, 46, 47] reporting on five trials did not specify any follow-up instructions. There were 12 articles [24, 25, 30, 31, 34, 35, 37-39, 41, 42, 45] comprising ten trials that provided participants with various physical activity guidance and/or support, including provision of exercise guidelines [24, 30, 31, 45], an exercise program [24, 30, 31, 34, 38, 39, 45], one-week inpatient stay [30], exercise booster sessions [35], various motivation and counselling sessions [24, 31, 37], and an invitation to join (or access to) a community gym or exercise program [24, 25, 31, 34, 38, 39, 45]. Five articles provided various follow up support or guidance via telephone calls [30, 37, 41, 42, 45] focused on general aftercare [30], motivation and health [37], reinforcing regular physical activity and preventing relapse [41, 42], and if multiple exercise sessions were missed [45].

Five of the 21 articles included in this review offered delayed care to the control group, comprising an identical exercise program during the follow-up phase [25, 32, 33], the ability to complete any exercise program available to them [47], or an individualised exercise program and referral [38].

Retention at follow-up

Retention was reported in 20 [24, 25, 29, 31-47] of the 21 articles. In articles that reported dropouts, combined intervention and control group dropouts ranged from 8.5% [42] to 57% [38] (mean= 24.4%). In groups that received an exercise intervention dropout ranged from 5% [42] to 65% [44] (mean= 24.5%), and in the control groups dropout ranged from 7% [39] to 58% [38] (mean= 26.8%) (see Table 2).

Physical activity measurement

Methods of measuring physical activity are described in Table 1. Of the 21 articles, 17 [24, 25, 29, 30, 32, 34, 36-42, 44-47] used self-report physical activity questionnaires, three [31, 35, 43] used device-based measures of physical activity, and one [33] did not report the method used to obtain physical activity levels. Of the 21 articles included in this review, 18 [24, 25, 29-32, 35-39, 41-47] reported either raw physical activity values or change values for both the control and intervention groups at follow-up. These data were reported using the percentage of participants meeting guidelines [24, 25, 32, 33, 37], MET-min/week [29, 30, 45], MET-h/week [40, 44, 46], moderate-to-vigorous minutes per week [31, 34, 43, 47], leisure time minutes per week [36, 38, 39, 41, 42], and activity counts per minute [35].

Self-report measures: multi-item recall questionnaires

The Godin Leisure-Time Exercise Questionnaire (GLTEQ) [60] was implemented by three [32, 34, 44] of the 21 articles. Modifications were made to the GLTEQ in two articles, with Courneya et al. [32] altering the GLTEQ to ask participants to recall a typical week over the past six months and Gaskin et al. [34] altering the examples to be more relevant to typical Australian activities. All three articles that utilised the GLTEQ [32, 34, 44] added a duration component to each item to calculate the total volume of physical activity performed from each intensity. Baumann et al. [30] used The Freiburg Questionnaire on Physical Activity (FFkA, German version) [70] in their trial. Two articles [25, 47] used standardised questions on frequency, duration and intensity of walking, cycling, and other exercise over a usual week in the month preceding, adapted from the Short Questionnaire to Assess Health-Enhancing Physical Activity (SQUASH) [61]. Kong et al. [36] utilised the Global Physical Activity Questionnaire (GPAQ) [71] to measure leisure-time physical activity. Physical activity recall was also collected in two articles [41, 42] via the Seven-day Physical Activity Recall (7-day PAR) [72] and a further two articles [38, 39] using the Scottish Physical Activity Questionnaire (SPAQ) [73]. Two articles [29, 45] used the International Physical Activity Questionnaire (IPAQ) [74], with Schmitz et al. [45] using the IPAQ to measure physical activity outside of the RET intervention at baseline and 12-month follow up.

Self-report measures: single-item recall questionnaires

Møller et al. [37] collected physical activity data using a self-developed questionnaire that measures leisure-time physical activity by asking participants to classify their physical activity levels as sedentary; walking, or cycling for pleasure; regular exercise at least three hours per week; or intense exercise for more than four hours per week [75]. Mijwel et al. [24] used a single item questionnaire that asked participants to categorise if they were or were not, currently meeting the exercise recommendations (defined as at least 150 minutes of moderate-intensity exercise per week).

Self-report measures: prospective reporting

Penttinen et al. [40] and Vehmanen et al. [46] reported on a single trial and assessed physical activity using a prospective two-week physical activity diary. In the diary, activities were categorised as light, moderate, vigorous, or very vigorous intensity [76].

Device-based measures: accelerometry

Three articles [31, 35, 43] used device-based measurements of physical activity; all three used accelerometry (Actigraph). All articles reported that participants were instructed to wear the accelerometer during all waking hours over a 7-day period, excluding during water-based activities. Bolam et al. [31] and Salerno et al. [43] analysed data using standardised cut-off points and wear time criteria [77], with final data reported as moderate-to-vigorous physical activity based on the average of the days with valid wear time (minimum 3-days, maximum 7-days). Kamshoff et al. [35, 78] converted the accelerations into activity counts per minute; no further details on analysis were described.
